# Support for Older People With Vision Impairment: A Rapid Realist Review

**DOI:** 10.1007/s44402-026-00125-0

**Published:** 2026-06-11

**Authors:** Anju Vaidya, Mark Davies, Carolyn Wallace, Pippa Anderson, Mari Jones, Bablin Molik, Rachel V. North, Barbara Ryan, Fiona Verity, Jennifer H. Acton

**Affiliations:** 1 School of Optometry and Vision Sciences, College of Biomedical and Life Sciences, Cardiff University, Cardiff, UK; 2 Faculty of Life Sciences and Education, University of South Wales, Pontypridd, UK; 3 Swansea Centre for Health Economics, SCHE, Faculty of Medicine, Health and Life Sciences, Swansea University, Swansea, UK; 4 Sight Cymru, Pontypool, UK; 5 Department of Health Sciences, College Health, Medicine and Life Sciences Brunel University of London, London, UK

**Keywords:** Aged, Eye, Public health, Realist synthesis, Social support, Vision loss, Visual impairment

## Abstract

**Background:**

Vision impairment among older adults is a significant public health issue impacting their physical and mental health and overall quality of life. However, access and navigation of support services are inconsistent and lacking. Recognising the need to gain insight to improve access to services in this population, this review aimed to understand how, why, for whom, to what extent and in what context the provisions for care and support for older people with vision impairment worked.

**Methods:**

A rapid Realist review was undertaken. Six databases (Cinahl, Cochrane Library, PubMed, Social Services Abstracts, Sociological Abstracts and Sociology Collection) were searched. The review followed the RAMESES Quality Standards for Realist Synthesis and retrieved documents were screened systematically to identify relevant papers. The papers were analysed through iterative and simultaneous use of coding, consolidating and conceptual mapping process to develop a Realist programme theory. Project Advisory Group members contributed to the processes. The Realist review was conducted from July 2023 to July 2024.

**Results:**

Ninety-one documents were reviewed and analysed. Novel insights into the provisions of care and support for older people with vision impairment were presented through seven interconnected components: Developing condition literacy, acceptance of the condition and readiness for help, timeliness, access to services, relationships, positive care trajectory and professional knowledge. All contributed to the development of the Programme Theory.

**Conclusion:**

The Programme Theory explained why and for whom the services worked, while limited information was found in terms of how and when the services were accessed. The key components identified around the Programme Theory were complex and interconnected in nature, underscoring the importance of a systems approach to effective service provision for older adults with vision impairment. Literature gaps were identified for further exploration in a Realist evaluation.

Key Points

- The study presents a rapid Realist review in which an interim Programme Theory was developed to understand how, why, for whom, to what extent and in what context the provisions for care and support for older people with vision impairments worked.
- Numerous and interconnected factors exist, including condition literacy, acceptance of the condition and readiness for help, timeliness, accessibility of services, relationships, positive care trajectory and professional knowledge. Novel insights reveal the nature of these interconnected factors and that a systems-based, person-centred approach is essential to improving service engagement, emotional well-being and overall care outcomes.
- Person care implications highlight the importance of an integrated systems approach to address the complex and evolving needs of this vulnerable population.

## Introduction

The World Health Organization (WHO) estimates that at least 2.2 billion people have vision impairment worldwide [1]. Most (90%) reside in low- and middle-income countries and over 90% could be prevented or treated with cost-effective interventions [2]. Vision impairment among people aged 60 years and above is a significant public health issue [3]. It impacts everyday life and social connections, regardless of its severity [4–6] by affecting their physical and mental health [6, 7], independence level [8], physical activity [9–13] and quality of life [14–16]. Older people with vision impairment are at high risk for chronic illness [17], falls [18, 19] and social exclusion [20]. Nevertheless, primary prevention strategies targeted at older adults neglect to account for many disorders, including vision impairment [21].

To be effective, social care, alongside other third sector (i.e., non-profit, voluntary, charity and community-based organisations) and healthcare support for vision impairment sometimes requires major lifestyle adjustments, but can empower older people to maintain independence and social connections [22, 23]. Yet access and navigation of social care support is problematic for many older adults with vision impairment [24] and their caregivers [25]. Evidence indicates that 41% of those with certifiable vision impairment were not certified [26], meaning this trigger for care and support can be missed. Organisational issues may prevent those being discharged from secondary care from receiving information about services they could benefit from, leaving access to services dependent on the initiative of the individual [27] and their support network. Additionally, a mismatch between the mental health needs of people with vision impairment and healthcare providers’ knowledge, skills and attitude has been reported [28].

Support and advice can be inconsistent and variable [29], and there is a lack of evidence-based guidance for clear pathways to navigate from primary care to social services, and support from third sector or community assets for older people with vision impairment. The aim of this review was to understand the provisions of care and support for older people with vision impairment, and how and when they are accessed. The type of support being reviewed included both services specific to those with vision impairment, as well as services for all older adults. Any relevant services were considered, for example, low vision rehabilitation, social care services, signposting and information provision, emotional support, community-based or peer support, discharge planning, transport assistance, problem-solving, digital skills, etc. The literature about support or care for older people with vision impairment is varied and complex, as it is not limited to one service or sector. The traditional methods used to evaluate interventions, particularly systematic reviews, focus predominantly on outcomes to understand what works [30]. Realist methodology was selected to understand how, why, for whom, to what extent and in what context the services for this population work, through understanding the impact of context and unravelling the underlying mechanisms and complex dynamics of these services [31].

Guided by Realist philosophy, a theory-based approach is adopted to understand the interaction between the contexts and underlying mechanisms leading to outcomes [32], which is appropriate to synthesise the evidence on complex social interventions.

A series of research questions grounded within Realist methodology was formulated as follows:

- How do older people with vision impairment access support or interventions aimed at them?
- When do older people with vision impairment access support or interventions aimed at them?
- For whom does the current provision of support for older people with vision impairment work or not?
- How and why do the support or intervention(s) work, and to what extent?

## Methods

Realist evidence was synthesised by unravelling the Context-Mechanism-Outcome configurations (CMOC) of the pathways from the literature, and developed an initial and interim Realist programme theory on support or care for older people with vision impairment via abductive reasoning and retroductive theorising [33]. The concepts and terms used in this review are described in Table 1.

**Table 1:** Key concepts in a Realist evaluation.

Concepts	Description
Context	Circumstances such as individual, institutional or environmental factors or dynamic features whose interaction with a mechanism makes the intervention work [46, 47].
Mechanism	A combination of resources (e.g., components of an intervention) and reasoning (e.g., response or perceptions of the participants) that generates intended or non-intended outcomes under the influence of specific contexts [31, 46, 157].
Outcome	Intended or non-intended effects are produced due to the interaction between context and mechanism [31].
Context–mechanism–outcome configurations (CMOC)	A statement describing how context triggers an underlying mechanism to produce an outcome [46].
Programme theory	A theory that explains how an intervention works [32, 37, 158]. An Initial Programme Theory is defined as an initial sketch of the programme theory describing how and why an intervention should work [37, 159]. As the Programme Theory evolves through development and testing, it may be referred to as an ‘interim’ and then a ‘refined’ Programme Theory.
Abduction	A ‘hunch driven approach’ that combines researcher’s intuition with common sense to obtain the most approximate explanation about underlying mechanisms triggered by contextual features, leading to theory generation [32, 46].
Retroduction	A process of theorising, in which a researcher starts with an intervention’s effect or outcome and works backward to identify the hidden mechanism that is necessary for the effect to be seen [46, 160].

This rapid Realist review was conducted between July 2023 and July 2024, based on our protocol (Prospero ID=CRD42023410721) [34] and principles put forward by Pawson et al. [35, 36] and Wong et al. [37, 38]. The review included iterative cycles of literature searches, screening, data extraction, theory generation and feedback from the research team members and stakeholders. An advisory group was formed that comprised 13 stakeholders from the voluntary organisations, professionals involved in delivering eye care services to people with vision impairment, lay members and people with living experience of vision impairment. The advisory group members were recruited through the professional and voluntary sector networks of the research team. The voluntary organisations were large and small charity-based organisations providing support for people with vision impairment in Wales, including peer support groups, independent living and advocacy support, eye clinic liaison and helpline support, campaigning and accessible information services. In addition, two independent academics with expertise in Realist methodology were invited to the group. These group members contributed to the refinement of search terms, databases, the development of the search strategy, developed CMOCs and programme theories.

This review followed six iterative steps [36, 39, 40] and Realist and Metanarrative Evidence Synthesis: Evolving Standards (RAMESES) guidelines [37, 38] and was informed by approaches used by other Realist studies [41, 42].

### Step 1: Identify the scope of the study

This Realist review was part of a wider project, a preventative approach for Access to a Sustainable, whole System pathway for older people with vISion impairmenT (ASSIST) study, aiming to understand how, why, for whom, in what context and to what extent the current provision of care and support for older people with vision impairment works in Wales. The scope and objective of this review are guided by the objective of the ASSIST study and have been developed to address the research questions. The review provides an opportunity to strengthen how to explain the evolving programme theory as part of the Realist evaluation through comparing national and/or international programme theories with local activities [39]. The Population, Intervention, Comparator and Outcome (PICO) framework was used to identify key concepts of the topic (Table 2).

**Table 2:** PICO framework.

Population	Adults over the age of 60 years with vision impairment.
Intervention	Any support aimed at helping older adults with vision impairment with activities of daily living or to maintain their independence.
Comparator	No comparator.
Outcome	The way the interventions support older people with vision impairment, how and for whom. The reason for interventions not being able to support older people with vision impairment.

### Step 2: Search for evidence

#### Development of search strategy

Initially, an informal scoping of the literature was undertaken for familiarisation purposes, supported through discussion with the research team and advisory group members before developing a formal search strategy. In view of the complex nature of the interventions for older people with vision impairment, the team members decided to develop and refine the programme theory based on the review findings. This process informed development of an initial programme theory (see definition in Table 1).

#### Data sources and search strategy implementation

**Fig. 1:**
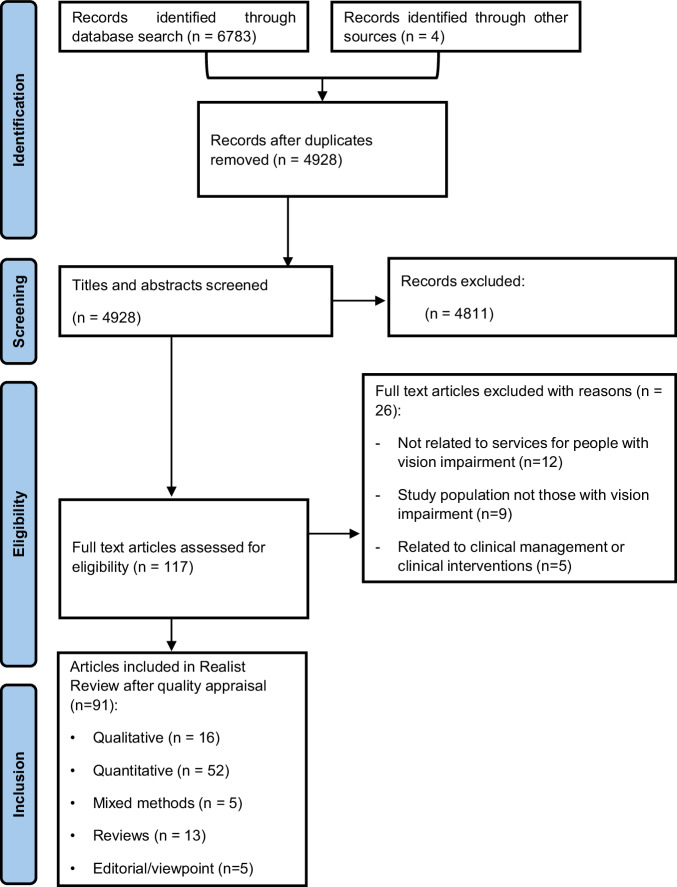
Literature search and screening process.

Six databases (CINAHL, Cochrane Library, Pubmed, Social Services Abstracts, Sociological Abstracts, Sociology Collection) were searched for documents related to support or interventions for older people with vision impairment, published between 1 January 2013 and 30 December 2022 (Fig. 1). Grey literature obtained from professional, local authority and voluntary sector websites from the UK were additionally analysed and further documents were provided by the project advisory group. The search strategy and inclusion and exclusion criteria are listed in Tables 3 and 4. A formal literature search in the databases yielded 6783 results (Fig. 1). The search results were exported and deduplicated in Zotero software (Corporation for Digital Scholarship, zotero.org) and extracted to Rayyan software (Rayyan Systems, Inc., rayyan.ai) for the researchers to validate the screening, which is discussed in Step 3.

**Table 3:** Search terms.

Concept 1: Vision impairment	(vision OR visual OR sight* OR “functional vision” OR “visual acuity” OR “visual field”) AND (low OR poor OR partial* OR impair* OR loss OR disorder* OR disabilit* OR threatening OR blind*)
Concept 2: Older adults (aged 60 years and above)	(Aged OR ageing OR elderly OR “older adult*” OR geriatri* OR carer OR mature OR “older person” NOT young NOT “non elder*”)
Concept 3: Intervention	(service* or intervention* or support or access or provision* OR prevent OR Rehab* OR reabl* OR information OR advice OR advocacy OR enabl*)

**Table 4:** Inclusion and exclusion criteria for the study.

Inclusion criteria	Exclusion criteria
Participant’s age ≥60 years with vision impairment	Participant’s age <60 years
Published literature from 2013 to 2022	Published literature before 2013
All types of documents with full access	
Services provided to people with low vision	Drugs and medical-related interventions, Animal studies
English language	Other than the English language.
Grey literature (key policy and guideline documents)	
Studies with an evaluation component	

### Step 3: Study selection

#### Screening process

The documents derived from the search results were screened based on the inclusion and exclusion criteria (Table 4). Titles and abstracts were screened by author AV, with a random 10% screened by author JA to check for consistency in screening application. Any disagreement was reviewed by author MD and resolved via discussion. The full text of 117 documents was then screened for eligibility (AV), with 10% double screened (JA) and disagreements reviewed (MD).

### Step 4: Quality appraisal

#### Rigour assessment

The full text screening of the 91 documents was undertaken with a focus on ‘relevance’ to the research questions and specifically, the degree to which the paper could advance the Programme Theory. Criteria to assess rigour of the papers were adopted based on guidance from the literature (Table 5) [37, 43, 44]. The methodology to assess rigour was based on a previously used framework in which rigour is described as the trustworthiness of the evidence, in which the credibility of the source is considered, as well as the appropriateness of the methods used [37, 43]. Rigour is further conceptualised by transparency of reporting [45]. Two graders (AV and JA) appraised the selected papers using the criteria to assess rigour (i.e., trustworthiness of the studies that meet the criteria) [43].

**Table 5:** Rigour assessment criteria.

Criteria	Trustworthiness
High	Good quality, assuredness in data collection, analysis and interpretation
Moderate	Contains enough description that provokes debate and discussion around data/information that indicates some form of assurance
Low	Poor quality, limited description, provokes doubt on interpretation

#### Rigour ratings

Documents were appraised against rigour categories: high (76), moderate (3) and low (12). Although 12 papers were scored with low rigour, all of the documents were included in the study, given the possibility of relevant information, which was further corroborated by other documents.

### Step 5: Data extraction

#### Data extraction framework

One researcher (AV) extracted the data from the 91 documents using a bespoke data extraction spreadsheet in Microsoft Excel (Microsoft Office, version 18.2503.12711.0, Microsoft.com), including these characteristics: authors, date, methodology, type of intervention, potential themes and substantive theory.

#### Coding

Full-text documents were also extracted to NVivo (version 12, QSR International, qsrinternational.com) for coding and development of CMOCs. The Realist CMOCs heuristic tool was used during the data coding and analysis to configure contexts (C), mechanisms (M) and outcomes (O) [31, 46, 47].

### Step 6: Data synthesis

#### Analytical approach

The review used the analytical tools, coding and consolidating as two key techniques to synthesise CMOCs and programme theories [48, 49]. Elements or patterns within the data were coded into broad ‘buckets’, by two researchers (JA and AV), before being explored further with reference to generative causation.

#### Development of initial programme theory

**Fig. 2:**
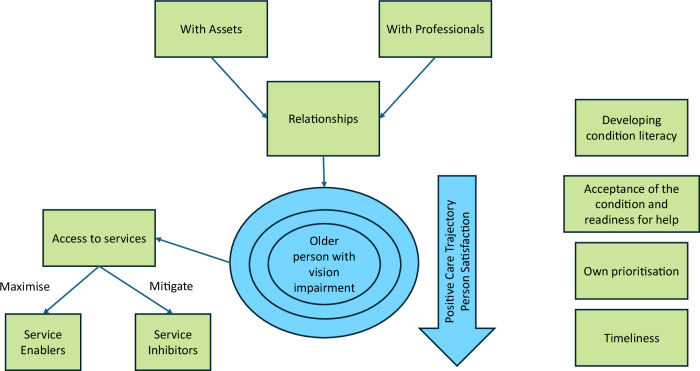
Initial programme theory. The term ‘assets’ is used to describe community-based resources, e.g., social groups, physical activities, arts, volunteering and support services, which older adults can engage with to improve their wellbeing and social connectedness.

The coded data extracts were used to build and then confirm, refute or refine the generated CMOCs. Two hundred and seventy-one CMOCs were generated from the full text document review, from which causal mechanisms were elicited retroductively and with abductive reasoning [50]. Similar CMOCs were then consolidated and grouped together using Realist logic under 69 common components of the emerging Programme Theory. Conceptual mapping [48] was applied to link CMOCs together, which were then synthesised and configured into eight overarching components, contributing to the development of the initial programme theory (Fig. 2).

#### Development of the interim programme theory

The coding, consolidating and conceptual mapping process occurred simultaneously and iteratively during the development of CMOCs and refinement of the programme theory via discussion with the review team and advisory group members [48]. Two research team members (MD and CW) supported the process and were involved in discussions related to the coding, development of CMOCs, theorising phases and synthesis.

**Fig. 3:**
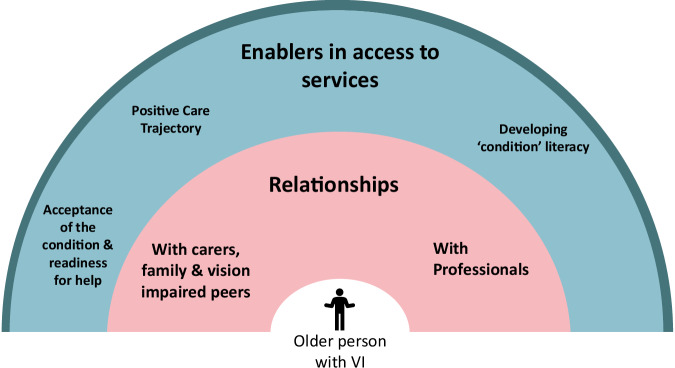
Interim programme theory. The conceptual model shows an older person with vision impairment (VI) at the forefront. The pink semi-circle represents the relationship component with carers, family, peers and professionals. The blue semi-circle represents the access to services component, which includes enablers that influence whether and how services are accessed.

A model (Fig. 3) explicating the support pathway for older people with vision impairment was developed further based on the Realist programme theory of both causation and implementation.

## Results

**Fig. 4:**
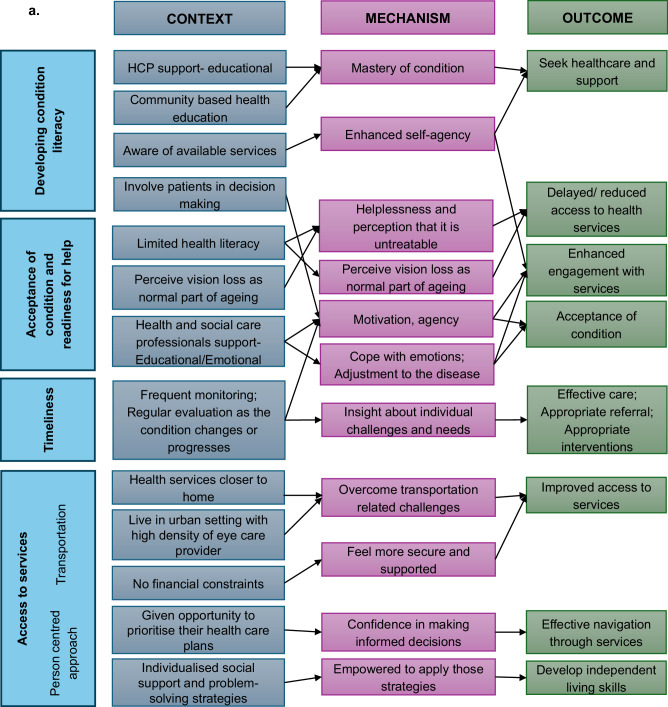

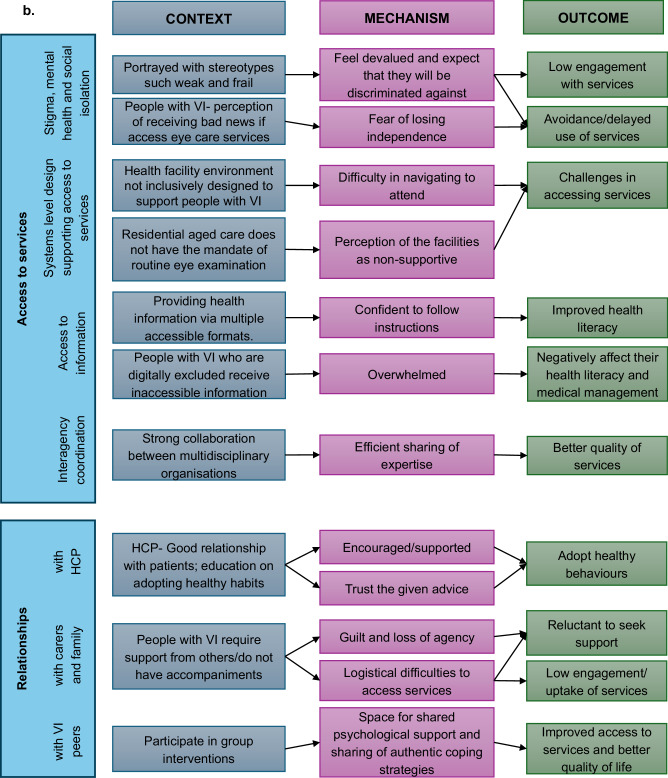
**a** Visual representation of Context-Mechanism-Outcome configurations (CMOC) and interim programme theory. HCP healthcare practitioner, VI vision impairment, GP general medical practitioner. **b** Visual representation of Context–mechanism–outcome configurations (CMOC) and interim programme theory. HCP healthcare practitioner, VI vision impairment.

The initial programme theory (Fig. 2) provided an explanation for the provisions of care and support for older people with vision impairment. The findings from the review were used to develop an interim programme theory (Fig. 3), consisting of the following components: *Developing condition literacy, Acceptance of the condition and readiness for help, Timeliness, Access to services, Relationships, Positive care trajectory and Professional knowledge*. The group of CMOCs contributing to each component was synthesised into one or two overarching CMOCs and presented in the narrative describing each component below. CMOCs within each component are represented visually in Fig. 4. The majority of studies were found in the healthcare domain; very few described social care or voluntary sector services.

### Developing condition literacy

*When older people with vision impairment develop “condition” literacy (C), their understanding and self-agency are enhanced (M), resulting in seeking engagement with eye/health care services (O).*

A total of 39 CMOCs derived from 26 papers contributed to this cross-cutting component on developing ‘condition’ literacy, which spans across other components in the Programme Theory and aligns with the sight loss journey of the individual (Fig. 3). For a patient, understanding their eye condition and the range of available services was a driver of engagement with health and social care. When healthcare professionals communicate information effectively about the individual’s eye condition and potential implications of utilising these services, it enhances their understanding, resulting in improved health literacy and increased motivation to pursue available services [51–58]. Community-based health education programmes similarly enhance eye health awareness in this population, fostering self-agency and increasing service utilisation [59–62]. This encourages a positive attitude and proactive approach and equips individuals to navigate services that best address their needs, leading to enhanced self-agency, service engagement and utilisation [55, 58, 61, 63–70].

Evidence also showed that enhanced health literacy improved the acceptability of referrals; for example, optometrists’ referrals to exercise programmes [51]. Involving individuals in shared decision-making was found to foster a sense of investment, resulting in improved treatment uptake [69]. In contrast, poor health literacy and a lack of insight into health services can trigger fear of the unknown, anxiety and mistrust, ultimately resulting in a lack of engagement with these services [54, 56–58, 61, 68, 71–74].

### Acceptance of the condition and readiness for help

*When older people with vision impairment are provided with support at the right time with respect to their readiness for help (C), it allows them to adjust to their loss and identify their needs (M), leading to acceptance of their condition and support (O).*

This component (supported by 10 documents and 10 CMOCs) focuses on the timing and manner in which older people with vision impairment accept or deny their condition and how this impacts their readiness to seek help. The findings revealed that many older people often deny their vision impairment due to limited health literacy, leading them to perceive vision loss as a normal part of aging and something untreatable. This denial can create helplessness and delay or reduce access to necessary services [61, 72, 75–77]. A study from Australia indicated that the grief associated with vision loss can lead to demotivation in engaging with services [78]. However, person-centred approaches, such as allowing time for individuals to adjust to their diagnosis [70]; identifying the individuals’ specific priorities and needs [79]; providing emotional support and offering education about their condition either early in the disease process or at the right time for the individual [53] can significantly enhance their readiness for change and motivation. Such strategies may support acceptance of the condition and increase willingness to seek help. It was reported that enhancing individuals’ awareness of their eye condition can strengthen their perception of the need for eye care, leading them to approach care providers with a more positive attitude, facilitating better interactions and care delivery [57].

### Timeliness

Seven documents and nine CMOCs were used to derive a component describing the timely management of older people with vision impairment through monitoring and follow-up by healthcare professionals [58, 60, 66, 75, 80–82]. Additionally, five documents and six CMOCs were used to describe the timely referral of older people with vision impairment [58, 60, 80, 83, 84].

*When older people with vision impairment receive frequent monitoring from healthcare professionals (C), their self-agency and motivation are enhanced (M), resulting in more effective care (O).*

Literature highlighted that repeated monitoring and follow-up substantially improved the quality of care and health outcomes. Repeated interactions with healthcare professionals enabled ongoing communication about the condition and problem-solving strategies, supporting the development of long-term goals and fostering independent living skills [66, 81, 82]. Such continuity of care appeared to motivate the uptake of recommendations and behaviour change, resulting in effective preventive care [58, 60, 75, 80].

*When healthcare professionals undertake effective clinical review of patients (C), they gain insight about the individual challenges (M), leading to identification of their needs and appropriate referral to specialist services (O).*

Frequent interactions between healthcare professionals and patients enable regular evaluation of the person’s condition as it progresses, helping to identify new challenges and needs. This approach allows healthcare professionals to invoke timely and tailored interventions based on the individual’s evolving needs [60, 80, 83]. Several studies highlighted that a thorough clinical review allows healthcare professionals to gain deeper insights into the person’s condition and needs, which helps them make appropriate referrals to specialist services, ensuring access to interventions that improve quality of life [58, 60, 80, 83, 84]. Early referrals to low vision care providers were found to foster rapport building and trust between the care provider and the individual, resulting in more effective, tailored interventions to address the individual’s specific needs [80, 84].

### Access to services

*When older people with vision impairment must face numerous and complex challenges (C), they experience negative emotions leading to loss of agency (M) and consequently, lack of engagement (O).*

A component describing access to services was derived from 57 documents and 102 CMOCs. Many complex enablers and inhibitors to access to services for older people with vision impairments were identified.

### Transportation

*When older people with vision impairment have better physical access to services, e.g., closer proximity of services and logistic support (C), they have increased capacity to overcome challenges around transportation (M), leading to engagement with and utilisation of the eye/health care services (O).*

Literature indicated that closer proximity of healthcare facilities to the homes of individuals enabled them to overcome transportation-related challenges such as travelling long distances, navigating unfamiliar locations and related costs, leading to improved access to services [55, 61, 65, 68, 71, 85].

Access to services was relatively easy for those in urban settings, given the high density of nearby and accessible care providers and healthcare facilities [54, 72, 74]. Conversely, some studies described access challenges presented by a low distribution of healthcare professionals and lengthy physical distance to the health facilities [73, 86, 87]. Development of teleophthalmology programmes may represent a solution, enhancing affordability and convenience in access [86, 88–90].

Additionally, literature indicated that being financially equipped can enable the affordability of transportation, making individuals feel secure and supported and facilitating service accessibility [54, 74, 77, 91]. In contrast, having financial constraints limited the affordability for travel or carer-related costs, leading to marginalisation and diminished service utilisation [54, 56, 57, 59–62, 68, 73–75, 81, 89, 92–97].

Positive examples of transportation systems included door-to-door services and community-based flexible facilities that consider individual needs, which enhanced accessibility through alleviating stress in managing journeys [70]. Conversely, weak public transport infrastructure limited the opportunity for individuals with vision impairment to travel, resulting in feelings of diminished independence or challenges to accessing services [53, 61, 62, 85, 86, 88, 98].

### Person-centred approach

*When older people with vision impairment are provided with individualised social support and trained in problem-solving strategies (C), they feel empowered to apply those strategies (M), resulting in better development of independent living skills (O).*

Fifteen CMOCs generated from 12 documents contributed to this component. Training for older people with vision impairment with problem-solving strategies and providing social support was found to empower them to apply those strategies and achieve their functional goals to overcome limitations in daily activities, which can also ameliorate symptoms of depression [99–103].

Managing multiple chronic conditions was found to be a barrier to timely access to services [75, 104, 105]. Some papers highlighted the complexity of the needs of older people with multi-sensory impairments, including hearing loss [85, 90, 106]. There is a need to acknowledge these complexities and provide ‘needs-based’ solutions. For instance, individualised and flexible rehabilitation approaches may include orientation and mobility training to enhance functional ability [85, 90, 106] and training to enhance skills in using assistive technologies [103, 107].

### Stigma, mental health and social isolation

*When people with vision impairment are portrayed with stereotypes such as ‘weak’ and ‘frail’ (C), they will feel devalued and expect to be discriminated against (M), leading to low uptake of services and avoidance of social participation (O).*

Five studies highlighted that the stigma associated with vision impairment led individuals to perceive the condition as a sign of weakness and frailty [68, 75, 94, 108, 109]. As a result, individuals were reluctant to seek support. Negative ‘weak’ stereotypes make individuals feel devalued and expect discrimination, which reduces their social participation and service utilisation [68, 75, 94, 108, 109]. Additionally, when individuals anticipated receiving bad news from healthcare professionals, they feared losing their independence due to the eye condition, which resulted in delays or avoidance in seeking services [68, 110, 111].

Several studies highlighted that when older people with vision impairment were socially isolated, they were more likely to develop depressive symptoms due to decreased self-esteem [58, 88, 112]. Studies underscored the contribution of emotional support in enhancing the individual’s mental health and self-agency, which improved coping behaviour over time and quality of life [58, 113].

### Systems-level design supporting access to services

*When eye care services are adequately commissioned to place the individual at the centre of their care (C), eye care needs of older people are recognised (M), resulting in access to services (O).*

With the continued demographic increase in the older adult population, the health and social care system faces unique challenges to provide care for these individuals who may have multiple medical co-morbidities, including eye conditions [114]. Those living in residential care were reported to face disparities in accessing eye care services, like ophthalmic screening, routine eye examinations and treatment [92, 104, 115, 116]. Reasons for such limited access include a lack of recognition of eye care needs along with associated time and cost constraints [92, 116, 117].

Some reports have demonstrated a lack of inclusiveness in the design of services affecting accessibility for individuals with vision impairment [92, 116, 118]. For example, when service design does not support the needs of those with vision impairment, such as a lack of accessible signage, voice call systems or seating priority, this leads to difficulties in navigating through services, resulting in inaccessible services [118].

*When screening for eye conditions and rehabilitation needs is embedded into service design for older people (C), people feel supported and motivated (M), which leads to their needs being addressed (O).*

Significantly reduced depressive symptoms were found in those with vision impairment who were screened for depression within eye care services and referred appropriately, as the individuals were able to receive support early and enhance their coping capacity [119]. Similarly, screening for eye conditions among older people in primary care facilitated early detection, timely management and appropriate referral [80, 92, 120].

The importance of integrated screening and care provision was emphasised for care home residents with vision impairment, to address their needs effectively [95, 116]. Another study emphasised the need to embed a support system for nursing staff in residential care homes, such as scheduling healthcare appointments and transportation facilities, particularly for older people with vision impairment, to facilitate access to services [92].

### Access to information

*When older people with vision impairment are provided with health information via multiple accessible formats (C), they feel confident to follow instructions (M), resulting in improved health literacy (O).*

A study of older individuals with vision impairment found significant challenges in accessing, understanding and using health information, with care providers often struggling to offer it in accessible formats [98]. Limited accessible options and poor awareness of available resources and rights led to frustration, dependence on family and a loss of privacy and independence [98]. Further barriers included digital exclusion, negatively affecting health literacy and medical management. The need for individuals to be digitally connected for health information and access to services, for instance via email, was found to be particularly challenging for older people with vision impairment who possessed limited digital skills [70]. Conversely, literature highlighted that providing individuals with vision impairment with timely health information in their preferred accessible format can enhance their understanding and adherence to any instructions, resulting in improved health literacy [69, 71, 98, 121, 122].

### Interagency co-ordination

*When there is good co-ordination between organisations with interdisciplinary engagement (C), it allows insight into scope of services and trust between agencies (M), resulting in appropriate referral and more targeted interventions (O).*

Effective care co-ordination was identified as essential for person-centred care. When care providers lacked inter-disciplinary engagement, there was limited understanding about each other’s roles and scopes of services, leading to misinterpretation around referral criteria. This resulted in hesitation to refer, ultimately leaving the needs of the individuals unmet [64, 70, 75, 78, 106]. However, strong collaboration between multiple disciplines or organisations can trigger efficient sharing of expertise on the needs of the individual, which may not only be limited to vision loss but also other co-morbidities, leading to improved quality of joined-up services [58, 66, 69, 81, 84, 87, 88, 106, 109, 123]. A study examining the potential for optometrists’ referrals to exercise programmes highlighted that good coordination and communication between community services and clinicians can lead to enhanced awareness and trust between agencies, resulting in more targeted interventions [51].

### Relationships

#### Relationship between healthcare professionals and older people with vision impairment

*When healthcare professionals have good relationships with people with vision impairment (C), individuals feel able to trust the professional advice (M) and feel encouraged to adopt healthy behaviours (O).*

Literature highlighted the importance of cultivating individuals’ confidence and trust in care providers, which influenced their engagement in services. Long-term and trusting relationships between individuals and their healthcare providers were found to be a powerful tool in fostering trust and confidence in the health and lifestyle recommendations. This resulted in successful behaviour modifications such as changes in diet and smoking habits and better treatment adherence [51, 80, 124]. A qualitative study further highlighted how having good relationships and supporting individuals to access services can enhance their knowledge and capacity to build connections and improve their readiness for change [79].

#### Relationship with carers and family

*When older people with vision impairment do not have family members or others to accompany them to healthcare appointments (C), they may face logistical difficulties/challenges in accessing services (M), resulting in them less likely to attend (O).*

A sub-component, derived from five papers, highlights the role of support from carers and family members in influencing an individual’s care-seeking behaviour. Older individuals with vision impairment and mobility restrictions were more likely to require assistance with tasks such as attending healthcare appointments and purchasing medicines [121, 125]. The need for accompaniment was identified as a significant challenge to utilising care services, particularly for those with fewer family members or those living only with a spouse, with less help to assist them [56, 57, 121, 126]. However, individuals receiving support from friends and family are able to overcome logistical challenges to navigate services better [70].

*When people with vision impairment require the support of others (C), it engenders feelings of guilt and loss of agency (M), resulting in a reluctance to seek support (O).*

For some individuals, requesting support from others to access services, participate in activities or attend groups was perceived as a burden on their carers. This perception often resulted in feelings of guilt and a sense of loss of agency, which in turn led to a lack of motivation to try new things or seek support [53].

*When healthcare professionals educate family members about the person’s health condition (C), they will gain a better understanding of the person’s capabilities and safety concerns (M), resulting in reduced overprotection from family members and greater independence of the individuals (O).*

Studies showed that individuals were often discouraged by family members to use vision rehabilitation strategies and services because of protectiveness and concern about safety, which limited the development of independent skills [75, 127]. Literature highlighted that educating the family members about the individual’s condition and capabilities may enhance their understanding and reduce overprotective attitudes [75, 127].

#### Relationship with vision-impaired peers

*When older people with vision impairment participate in group interventions, e.g., peer support groups where they meet others with similar living experience (C), this creates a space for shared psychological support and sharing of authentic coping strategies (M), leading to improved access to services and better quality of life (O).*

Eleven CMOCs generated from eight documents contributed to this sub-component that highlights the facilitative role of sharing relationships with peers with similar living experiences toward maintaining independence [51, 58, 70, 85, 90, 111, 128, 129]. The social environment of peer support groups [51, 85, 129] creates a safe space to share personal experiences of vision loss and related fears and frustrations. The opportunity to share experiences and realise that they are not alone was felt to enhance self-esteem, confidence and feelings of worthiness, which led to improved problem-solving skills in adapting to vision loss [58, 111]. Such groups were reported as a source of information, for instance, understanding local community services and ways to access them [70]. However, it should also be acknowledged that some may prefer individual rather than group settings for some activities, e.g., exercise programmes [51].

### Positive care trajectory

*When older people with vision impairment are provided with continuity- and relationship-based care (C), they feel comfortable in sharing their emotional problems (M), resulting in referral uptake and satisfaction (O).*

Person satisfaction was considered an outcome of a positive care trajectory. This component was derived from 10 documents [69, 75, 109, 118, 119, 122, 130–132]. Screening for depression within eye care services, with referral to the general medical practitioner (GP) was found to detect and treat depressive symptoms effectively in older people with vision impairment [119]. The continuity of care provided by GPs fostered trust and comfort in sharing emotional problems, resulting in patient satisfaction. However, people were less likely to seek referral to a mental health professional due to the stigma associated with seeking psychological support. Therefore, GP referrals were found to be an acceptable and less stigmatised way to seek support for depressive symptoms [119].

*When healthcare professionals communicate with empathy and consideration of an older person with vision impairment needs (C), the person feels valued and capacitated (M), leading to person satisfaction (O).*

Although strong communication is a pillar of person-centred care, for those with sensory impairments, communication challenges may result in misunderstanding of information, reduced treatment adherence, lower person satisfaction and negative health outcomes [75, 122, 130, 132]. Healthcare professionals faced challenges in communication and providing adequate care, given the additional time and effort required. A lack of training to support occupational therapists in effectively communicating with those with sensory impairments was identified [132]. To ensure high-quality person-provider communication, training care providers about the needs of those with sensory impairment is essential for effective exchange of information and improved health outcomes [109, 132].

Literature highlighted that when the health services provided sufficient consultation time and communicated with service users in a calm and empathetic manner, individuals felt valued and taken seriously. This communication behaviour can result in satisfaction, empowerment to participate in shared decision-making and co-management of their care trajectory [69, 75, 118, 122, 131].

### Professional knowledge

This component was derived from eight documents and focusses on approaches to improve the care trajectory of older individuals with vision impairment.

*When healthcare professionals are knowledgeable about vision rehabilitation and other support services (C), they feel confident in managing a person’s care trajectory (M), which facilitates a seamless multi-disciplinary cross-agency approach (O).*

Inter-professional training of care providers across disciplines can empower professionals to deliver services effectively and meet the diverse needs of individuals [51, 58, 94, 106, 122, 132]. The importance of prioritising a person’s needs across disciplinary boundaries was emphasised in the context of hearing, vision and cognitive impairments [106]. Cross-disciplinary training in using assessment tools, such as training memory specialists to conduct vision and hearing assessments, can enhance a professional’s confidence and competence in managing people effectively and creating cohesive care pathways across disciplines [106].

Training eye care providers to assess falls prevention in older people with vision impairment can raise their awareness of the risk of falls, enabling more tailored management and appropriate referrals [51]. A lack of specialist skills in assessing individuals with vision impairment can lead to diminished confidence in evaluating their needs and recognising their potential, ultimately resulting in insufficient support for these individuals [58, 122].

Embedding awareness of vision impairment into training for healthcare professionals has been emphasised. Such training should include addressing emotional aspects of vision loss, improving communication with individuals with sensory deficits and considering their needs when delivering information, such as providing large print materials and auditory cues [132, 133]. Addressing the emotional aspects of vision loss and communication techniques during training can help care providers become more aware and empathetic, to enhance person-provider communication, care quality and appropriate referrals [58, 132–134]. Moreover, this approach encourages healthcare professionals to be more mindful of individuals’ needs and improve their ability to provide information in accessible formats, ultimately making the healthcare services more inclusive [132].

## Discussion

This Realist review aimed to understand *how, when, for whom* and *why* the provisions of care and support for older people with vision impairment work. Services described in the literature reviewed included low vision and eye care services, vision rehabilitation, multidisciplinary healthcare, mental health support, social care services, community-based programmes, peer support, home/domiciliary care, falls-risk assessment, exercise programmes, problem-solving, digital skills, accessible health information provision, discharge planning and transport assistance. The Programme Theory developed from this review highlighted current knowledge of key areas for improving the care and support provisions, particularly in relation to identifying gaps in access and areas of best practice. The components identified were: (i) Developing condition literacy, (ii) Readiness/acceptance for help, (iii) Timeliness, (iv) Access to services, (v) Relationships, (vi) Positive care trajectory and (vii) Professional knowledge. The existing literature partially addressed the aims of the review, in that many of the components answered *for whom* and *why* support worked, but there was less information about how and when support was accessed. Specifically, the ‘*how*’ and ‘*when*’ were described by components on transport, systems level design, access to information and relationships and condition literacy, acceptance and timeliness, respectively. Complex and interconnected factors were evident in addressing the research questions and it was acknowledged that information may be location-specific and evolving with changing services over time. All of the components identified in the Programme Theory had relevance across different service domains, including healthcare, social services, mental health support and rehabilitation.

To understand for whom and why support worked, key findings indicated that those with good literacy and/or receiving the right support at the right time were more likely to engage with all types of services. Those provided with accessible health information were well supported to develop condition literacy. More affluent individuals, with good access to transportation, as well as those with strong family support, were better able to attend peer groups and other services. Individuals who were able to participate in group interventions gained the benefit of peer support and shared coping strategies. Those who received training in problem-solving skills, e.g., from the third sector, developed independent living skills more effectively. Persons having good relationships with health and social care professionals supporting them were enabled to follow health advice.

Key findings emerged from the components within the review, including numerous and complex enablers and barriers to accessing services such as transportation, the importance of accessible information and understanding of the condition and individuals’ relationships with their care providers, family and peers. Given the complex needs of individuals with vision impairment, which are not confined to a specific time point in their sight loss journey, a range of support services were identified, including eye care, rehabilitation, mental health support, peer groups, community-based programmes and exercise activities. A bespoke pathway for older individuals with vision impairment could not be identified, which highlights the complexities around needs and the importance of addressing the range of needs. Thus, this review presents a novel synthesis and emphasises the application of a systems approach to address the complex needs of this population effectively.

This review identified interconnected, continuous and ongoing issues related to individuals’ access to all types of services. These findings mirror previous studies, in which communication of information and education about a person’s condition and available services was central for motivation to seek or engage with services for older people with vision impairment [42, 135]. Although the review highlighted challenges and disparities in access to information, ensuring timely access to information about the condition and service pathways, in the preferred accessible format, is vital. Such access is considered to make the individuals feel empowered to absorb the information at their own pace and improve their health literacy, service engagement and treatment concordance [42, 136, 137]. Furthermore, the review highlighted that enhancing an individual’s condition literacy and involving them in decision making increased their sense of accountability, resulting in improved treatment uptake. This finding aligned with previous research assessing facilitators and barriers to person involvement in treatment decision making [42, 138]. Specifically, the need for the care provider to communicate clearly, empathetically and with respect was emphasised, which can lead to enhancing personal experience and comfort to participate in shared decision-making [42, 138]. Involving people in treatment decision-making was found to improve service engagement and treatment concordance [139, 140].

Toward understanding *why* support works, improving an individual’s understanding of their condition and service pathways was found to trigger a positive shift in attitude toward their condition, resulting in enhanced likelihood to accept their condition and readiness to seek and accept healthcare, home care and vision-related services [55, 58, 61, 63–70]. Conversely, in identifying *why support does not work*, prior to acceptance, grief associated with a diagnosis was described [141, 142]. In a state of grief, individuals may experience negative emotions such as stress and anxiety from the diagnosis and treatment information, demotivating them from engaging with low vision and rehabilitation services, counselling and peer support [141, 142]. To address this issue, allocating sufficient time is needed for the individuals to assimilate the given information [143].

In understanding *for whom* the support may or may not work, the review, in line with other studies [142, 144], highlighted that some individuals felt vision impairment was associated with negative stereotypes akin to being ‘frail’ or ‘devalued’. The stigma around the condition inhibited these individuals from disclosing their condition or expressing concerns to others, as well as from engaging with services and social activities [142, 144], leading to social isolation and mental health conditions like depression. Due to the stigma, the review findings highlighted that these individuals were less likely to accept referral to mental health professionals to uptake psychological support. However, a referral to the GP following positive screening for depression was more acceptable, particularly for those who had a trusting relationship with their GP. These findings are supported by another study [145], highlighting the importance of trusting relationships with the care providers for mental health service engagement.

The person-centred approach is widely recognised as a key element of high-quality care, in which individuals’ unique needs are identified and tailored support is provided to help them adjust to their condition [146, 147]. This approach emerged as a recurring and interrelated component in the review for understanding how and why support was effective. A number of papers highlighted strategies that identified individual’s needs, enhanced their readiness for change, motivation for service engagement, functional abilities and overall psycho-social wellbeing. These included training in problem-solving strategies, such as rehabilitation services, emotional support and education about vision impairment [147–151]. Furthermore, the review indicated education about the condition and support tailored to meet individual needs as key mechanisms in promoting acceptance of vision loss and improving overall well-being. Individuals in receipt of tailored information and guidance were more likely to engage with low vision and rehabilitation services, mental health support and exercise-based programmes, as well as feel motivated to participate in rehabilitation and experience improved emotional resilience [148, 150, 151]. Hence, by addressing both practical and emotional needs via person-centred strategies, care providers can help individuals with vision impairment achieve better outcomes and quality of life. The importance of effective person-provider communication was highlighted in the context of how and why support works, in addition to the need for adequate professional knowledge, which is supported by interdisciplinary training [51, 58, 94, 106, 122]. Such training is essential to empower care providers with the necessary skills to use effective communication techniques, understand individuals’ emotional states and deliver person-centred care. These findings aligned with previous studies [146, 152], which emphasised the importance of cross-disciplinary communication training programmes, particularly related to sensory impairments. However, a significant challenge identified in person-centred care is the increased consulting time required for effective communication. Studies suggest that longer consultations are often impractical due to time constraints, leading healthcare professionals to prioritise clinical components over person education. Nonetheless, with adequate training and practice, healthcare professionals can integrate person-centred communication more effectively into their practice [139, 153].

The review findings emphasised that, alongside interdisciplinary training, care co-ordination held a crucial role in delivering person-centred care and enhancing overall quality of care for older individuals with vision impairment. Effective coordination across disciplines or organisations facilitated seamless sharing of expertise, ensuring that individuals receive comprehensive and well-integrated support, particularly beneficial for addressing both vision loss and other co-morbidities [58, 66, 69, 81, 84, 87, 88, 106, 109, 123]. These findings are supported by various studies [154, 155], highlighting the contribution of enhanced knowledge amongst care providers and coordination efforts across disciplines on effective referrals, better health and wellbeing outcomes.

Physical access to services was a key issue identified in the review, in understanding how individuals access services. Those who resided near health facilities or in urban settings with better healthcare infrastructure were able to overcome transportation-related challenges, enhancing their access to services [54, 55, 72]. However, barriers included the inability to drive due to vision loss, which often led to feelings of reduced independence [53]. The reviewed studies focussed primarily on access to healthcare services, with limited evidence on how transportation challenges impacted daily activities and social participation.

### Strength and limitations

Notably, the studies included in the review were mostly limited to the healthcare domain. A smaller proportion of papers described services from social care or from the third sector aimed at supporting older people with vision impairment, mostly drawn from grey literature sources. Such predominance of healthcare-based evidence may limit the transferability of findings to non-health settings. While the included documents were heterogenous, such diversity is an inherent feature of Realist synthesis, which draws on pluralistic evidence to identify and test CMOCs. This enables transferable explanations to be developed from varied sources, but also means that the scope and depth of available evidence may vary. Additionally, the restriction to English-language sources may have resulted in the exclusion of other relevant perspectives. A Rapid Realist review was chosen to identify key mechanisms and contextual factors that influence outcomes in a complex area such as this [156]. This approach is more streamlined than a Realist Review and offers specific insights compared with a systematic review. Timeliness was crucial as this review was part of a wider study that included the development of a health economic model and qualitative research. Both of these work packages required the information from this review to inform their development. Strengths of the review include adherence to the RAMESES Quality Standards for Realist Synthesis [37] for reporting and the use of a Project Advisory Group, which included professionals, lay members and those with living experience who reviewed the literature search terms and the development of the programme theory. The review formed part of a wider Realist evaluation and the literature searches were undertaken within a defined timeframe, consistent with rapid evidence synthesis approaches.

## Conclusion

In conclusion, the literature partially addressed the Realist research questions, answering for whom and why support worked, but providing less detail on how and when it was accessed. Information was identified encompassing a range of complex and interconnected factors such as transport, system design, accessibility of information, relationships with others, condition literacy and timeliness of support. This gives a partial representation of systems and support available. The components identified appear to bear relevance across different service domains, including healthcare, social care, mental health support and rehabilitation. Their breadth suggests opportunities for system-level improvement; however, further research is needed to differentiate how these components operate within specific service contexts.

Overall, the findings highlighted that there is no one-size-fits-all approach to improve access to services for older adults with vision impairment due to their diverse needs at different stages. Therefore, a holistic approach that encompasses different interconnected factors and employs a person-centred approach, such as tailored education, rehabilitative and emotional support can make the services more effective and improve the overall wellbeing of this population. Understanding the synergy between the key components can help identify their implications for a systems approach, underscoring the importance of further studies on their synergistic functioning. Furthermore, although social services are crucial in improving the quality of life of individuals, evidence found in the review was inadequate and needs further exploration. The review is part of a wider project in which the interim programme theory was tested further to understand the current provision in a national population and how services and systems may be improved to support this vulnerable population better. The impact on personal, economic, health and care service outcomes of improvement of access to support, from social services and the third sector, is an issue requiring further research.

## Data Availability

No datasets were generated or analysed during the current study.
